# The mitochondrial genome of a deep-sea oplophoroid of the genus *Acanthephyra* (Crustacea: Caridea: Oplophoroidea)

**DOI:** 10.1080/23802359.2020.1831995

**Published:** 2020-11-03

**Authors:** Peng Xu, Xiangxing Gao, Xiaogu Wang

**Affiliations:** a Key Laboratory of Marine Ecosystem Dynamics, Second Institute of Oceanography, Ministry of Natural Resources, Hangzhou, P. R. China; b National Deep Sea Center, Qingdao, P. R. China

**Keywords:** Decapoda, mitogenome, phylogeny, South China Sea

## Abstract

The genus *Acanthephyra* mainly inhabits deep waters with the maximum depth exceeding 5000 m. It has a wide distribution, except in high latitude areas. Here, we report the mitochondrial genome of *Acanthephyra* sp. which was collected from the northeast of South China Sea. The genome is 16,205 bp in length with a 61.52% AT content. It contains 13 protein-coding genes (PCGs), 2 ribosomal *RNA* genes, and 22 transfer *RNA* genes. Phylogenetic analysis shows that the present species is closest to *A. smithi* and Oplophoroidea has a close relationship with Bresilioidea in Caridea.

The genus *Acanthephyra* (Milne-Edwards, 1881) comprises 27 species and is considered to be existing in the meso- and bathypelagic zones of the oceans (Kemp 1939; Crosnier and Forest 1968, 1973; Milne-Edwards 1881; Smith 1885; Chace 1986; Bate 1888; De Grave and Fransen 2011). Most species of the genus have morphological adaptations to pelagic lifestyle, such as natatory exopods and slightly calcified carapace (Bauer 2004; Cardoso 2013). In this study, the complete mitochondrial genome from a deep-sea *Acanthephyra* sp. was described, and its relationship with close related species was investigated.

The specimen was collected from the northeast of South China Sea (117°55′42″E, 20°59′43″N, 1302 m depth) and was identified as *Acanthephyra* sp. based on its morphological characters. This species is considered to be a new species and will be named within the next six months. The specimen (SRSIO19050306) and its DNA (DNASIO19050306) are deposited in the Sample Repository of the Second Institute of Oceanography, Ministry of Natural Resources, Hangzhou, China. DNA was extracted with QIAamp Tissue Kit (QIAGEN, Hilden, Germany) and mitochondrial DNA was amplified with a DNA REPLI-g Mitochondrial DNA Kit (QIAGEN, Hilden, Germany) as directed by the manufacturer. Library construction and sequencing were performed by Biozeron (Biozeron, Shanghai, China) using the Illumina Hiseq4000 sequencing platform (Illumina, San Diego, CA).

The complete mitogenome sequence of *Acanthephyra* sp. is 16,205 bp in length with a 61.52% AT content. It contains 13 protein-coding genes (PCGs), 2 ribosomal *RNA* genes, and 22 transfer *RNA* genes. Among the 37 genes, both *rRNA* genes (*rrnL* and *rrnS*) are encoded on the light strand, as in the other crustacean mitochondrial genomes. Eight *tRNA* genes (*trnC-tgc*, *trnF-ttc*, *trnH-cac*, *trnL1-cta*, *trnP-cca*, *trnQ-caa*, *trnV-gta*, and *trnY-tac*) are encoded on the light strand. Only four PCGs (*nad1*, *nad4*, *nad4L*, and *nad5*) are encoded on the light strand, whereas the other nine PCGs are located on the heavy strand. Five PCGs (*atp8*, *nad2*, *nad3*, *nad4*, and *nad4L*) are initiated by ATT. Three PCGs (*cob*, *cox2*, and *cox3*) are started by ATG. Three PCGs (*atp6*, *nad1*, and *nad5*) are started by ATA. The remaining two PCGs (*cox1* and *nad6*) are initiated by ATC. Nine PCGs (*atp6*, *nad5*, *nad6*, *cox2*, *cox3*, *atp8*, *nad2*, *nad3*, and *nad4L*) terminate with the typical TAA as stop codon, while three PCGs (*nad1*, *cox1*, and *cob*) end with TAG. Only one PCG (nad4) terminate with the abbreviated stop codon T. A total of 22 transfer *RNA* genes range in size from 60 to 73 bp. The mitogenome of *Acanthephyra* sp. has been deposited in GenBank under accession number MT879756.

In order to explore the phylogenetic relationship of *Acanthephyra* sp. with the other shrimps, a total of 17 mitochondrial genomes from Decapoda were obtained from the GenBank database. The mitochondrial PCGs were used for phylogenetic analysis by maximum likelihood (ML) method. In the phylogenetic tree, *Acanthephyra* sp. was closely related to *A. smithi*, and they clustered with *Oplophorus typus* into a branch. Meanwhile, Oplophoroidea had a close relationship with Bresilioidea in Caridea (Figure 1). In this study, we present the complete mitochondrial genome sequence of *Acanthephyra* sp., which would contribute to further comparative mitogenome studies of Caridea. Besides, more mitochondrial genomic data and further analysis are required to reveal phylogeny and evolution of shrimps.

**Figure 1.**
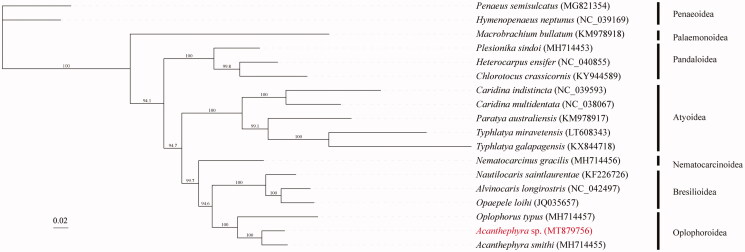
Phylogenetic tree of *Acanthephyra* sp. and other mitogenomes from Decapoda based on mitochondrial PCGs.

## Data Availability

All sequences generated or used in this study are deposited in NCBI GenBank (www.ncbi.nlm.nih.gov/) and the accession numbers are detailed in Figure 1.
